# Porous Nano-Fiber Structure of Modified Electrospun Chitosan GBR Membranes Improve Osteoblast Calcium Phosphate Deposition in Osteoblast-Fibroblast Co-Cultures

**DOI:** 10.3390/md22040160

**Published:** 2024-03-30

**Authors:** Hengjie Su, Tomoko Fujiwara, Omar Skalli, Gretchen Schreyack Selders, Ting Li, Linna Mao, Joel D. Bumgardner

**Affiliations:** 1 Institute of Biomedical Engineering, Chinese Academy of Medical Sciences & Peking Union Medical College, Tianjin 300192, China; 2 Department of Biomedical Engineering, University of Tennessee Health Science Center-Memphis Joint Graduate Biomedical Engineering Program, The University of Memphis, Memphis, TN 38152, USA; 3 Department of Chemistry, The University of Memphis, Memphis, TN 38152, USA; tfjiwara@memphis.edu; 4 Integrated Microscopy Center, The University of Memphis, Memphis, TN 38152, USA

**Keywords:** chitosan, electrospinning, guided bone regeneration, calcium phosphate deposition, co-culture

## Abstract

Desirable characteristics of electrospun chitosan membranes (ESCM) for guided bone regeneration are their nanofiber structure that mimics the extracellular fiber matrix and porosity for the exchange of signals between bone and soft tissue compartments. However, ESCM are susceptible to swelling and loss of nanofiber and porous structure in physiological environments. A novel post-electrospinning method using di-tert-butyl dicarbonate (tBOC) prevents swelling and loss of nanofibrous structure better than sodium carbonate treatments. This study aimed to evaluate the hypothesis that retention of nanofiber morphology and high porosity of tBOC-modified ESCM (tBOC-ESCM) would support more bone mineralization in osteoblast-fibroblast co-cultures compared to Na_2_CO_3_ treated membranes (Na_2_CO_3_-ESCM) and solution-cast chitosan solid films (CM-film). The results showed that only the tBOC-ESCM retained the nanofibrous structure and had approximately 14 times more pore volume than Na_2_CO_3_-ESCM and thousands of times more pore volume than CM-films, respectively. In co-cultures, the tBOC-ESCM resulted in a significantly greater calcium-phosphate deposition by osteoblasts than either the Na_2_CO_3_-ESCM or CM-film (*p* < 0.05). This work supports the study hypothesis that tBOC-ESCM with nanofiber structure and high porosity promotes the exchange of signals between osteoblasts and fibroblasts, leading to improved mineralization in vitro and thus potentially improved bone healing and regeneration in guided bone regeneration applications

## 1. Introduction

Guided bone regeneration (GBR) membranes are used in dental applications to prevent the invasion of gingival tissues into the bone-regenerating compartments. In addition to the shielding/barrier function of the membranes, the interconnected porosity and structure of the barrier membranes are reported as also playing important roles in healing by enabling an exchange of nutrients and growth factors between the gingival and bone compartments and mimicking extracellular matrix structure of newly forming tissues [1,2,3,4,5]. Electrospinning is one method to make GBR membranes that mimic the native nanofibrous extracellular matrix (ECM) structure and provide an interconnected porosity to allow the diffusion of nutrition, metabolites, and soluble factors between compartments that support healing [6]. 

Chitosan is a natural polysaccharide that has been widely researched as a GBR material due to its biocompatibility, homology to native ECM component hyaluronan, degradability, and ability to be electrospun [7]. However, electrospun chitosan membranes can be highly susceptible to swelling and loss of nanofibers and the interconnected porous structure [8,9]. Our group has successfully developed a novel post-electrospinning method that retains the nanofibrous structure of electrospun chitosan membranes (ESCM) better than other methods based on alkali treatments [7]. The post-spinning modification involves first removing acetate ions left over from the electrospinning process in the fibers using triethylamine (TEA) and then reacting di-tert-butyl dicarbonate (tBOC) with the chitosan amino groups to create a hydrophobic wrap on the outside of the electrospun chitosan fibers that prevents fiber swelling [7]. We demonstrated that the TEA/tBOC modified ESCM (TEA/tBOC-ESCM) exhibited little swelling and retained their nanofiber structure in physiological solutions, whereas a typical Na_2_CO_3_ treatment used to remove acetate ion salts resulted in extensive swelling and loss of nanofiber structure. The TEA/tBOC-ESCM were shown to be compatible with cultured cells, to degrade in vitro, to possess adequate mechanical properties for GBR applications, to provide effective barrier function, and to support bone healing in a rodent model. 

This study aimed to further examine the hypothesis that the retained nanofiber morphology and porosity of the TEA/tBOC-ESCM improves in vitro cell performance as compared to Na_2_CO_3_ treated ESCMs (Na_2_CO_3_-ESCM) and solution-cast chitosan solid film membranes (CM-film). The membranes were characterized for surface morphology, porosity, water contact angle and ash and endotoxin content. Fibroblast and osteoblast cells in individual and co-cultures were used to evaluate the effect of nanofiber structure and porosity on cell growth as well as osteoblastic mineralization. The co-culture model was introduced to simulate the clinical application of GBR membrane treatments that aimed to separate and prevent the ingrowth of fibroblasts into sites of osteoblast growth and mineralization.

## 2. Results and Discussion

### 2.1. Membrane Characterizations

**Figure 1:**
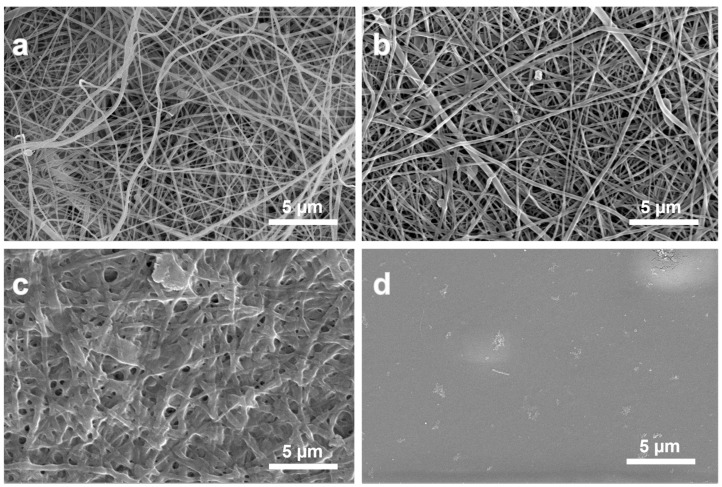
SEM images of the (**a**) non-treated chitosan electrospun membrane, (**b**) TEA/tBOC-ESCM, (**c**) Na_2_CO_3_-ESCM, and (**d**) the CM-film. The TEA/tBOC-ESCM exhibited similar nanofiber morphology and diameters as the as-spun membranes, whereas the Na_2_CO_3_-ESCM showed significant fiber swelling and loss of nanofiber morphology. The CM-film exhibited a smooth surface and no fibrous structure.

Results for the characterizations of the test membranes for fiber structure and diameter, porosity, hydrophobicity endotoxins and ash content are shown in Figure 1 and Table 1. The fiber diameter of the non-treated ESCM was 172 ± 48 nm, while that of the TEA/tBOC-ESCM was 219 ± 93 nm. There were no obvious fibers in the Na_2_CO_3_-ESCM, and no discernible structure in the CM-film. Consistent with previous reports, SEM microphotographs and analysis revealed that only the TEA/tBOC-ESCM were composed of well-defined nanometer diameter fibers, whereas the Na_2_CO_3_-ESCM exhibited significant fiber swelling, making the determination of individual fiber diameters difficult [7,10]. Because of the retained nanofiber structure, the TEA/tBOC-ESCM exhibited approximately 14 times greater porosity than the Na_2_CO_3_-ESCM (Table 1). As the CM-films were produced using a solution casting method, they lacked both fiber structure and significant porosity. The porosity of the GBR membranes is reported to be important for allowing nutrient and cell signaling exchange between osteogenic and soft/epithelial tissue compartments [11,12,13,14]. The significantly greater porosity of the TEA/tBOC-ESCM due to the retention of the nanofiber structure may be an advantage for improved nutrient and signal exchange as compared to the lower porosity Na_2_CO_3_-ESCM or essentially non-porous CM-film. 

There was no significant difference in the median pore size of the two ESCM membranes, and even the solution-cast CM-film exhibited a range of pore sizes (Table 1). Since the CM-film becomes extremely brittle and stiff after neutralization, the small pores in the CM-film were attributed to minor defects that may arise in the casting, drying and neutralization of the films and or in manipulating films using forceps. The similarity in pore size between the TEA/tBOC-ESCM and Na_2_CO_3_-ESCM is more indicative of spaces between the randomly oriented fibers. Nevertheless, the range of pore sizes (~5 to ~44 μm) of both ESCMs is similar to other studies of electrospun membranes for GBR applications [11,12,13,14]. These studies report that these small pore sizes and the circuitous connections of the pores due to non-woven random fiber networks limit cell migration through the membranes, which is important to their barrier function while also allowing for nutrient and signal exchange. 

The CM-film had the statistically highest water contact angle, followed by the TEA/tBOC-ESCM and then the Na_2_CO_3_-ESCM, which had the statistically lowest contact angle (Table 1). The high hydrophobic character of the CM-film is attributed in part to the strong alkali treatment, which deprotonates the amino groups in the chitosan polymer in addition to neutralizing and removing acetate ions from the solution casting process. The increased hydrophobicity of TEA/tBOC-ESCM stems from the methyl groups within the tBOC group, contrasting with Na_2_CO_3_-ESCM, which retains residual carbonate salts contributing to its previously reported higher hydrophilicity [7]. Although hydrophobic biomaterial surfaces are often linked to reduced cellular attachment and spreading, this may not pose a significant concern for GBR applications, where the primary role is to serve as a barrier against cellular migration. However, due to the gradual hydrolysis of tBOC from the ESCM over a period of 7–10 days, and given that the membranes feature pores within the range of 40μm in diameter, which is sufficient for tissue ingrowth, the integration of the membrane into local tissues does occur [7,15,16]. The integration of the membrane within tissues is advantageous for stabilizing its placement and guiding tissue growth during healing.

Membranes were tested for endotoxin using a Limulus Amebocyte Lysate (LAL) based assay (Table 1). Endotoxin is a component of the outer cell wall of Gram-negative bacteria and may persist as a contaminant in chitosan powders after processing from the original biological source material and impact cellular and host tissue responses such as inflammation and or sepsis [17]. The results indicated that the endotoxin concentrations of the membranes were very low, remaining below the 0.1 EU/mL test kit limit. This value is also beneath the 0.5 EU/mL limit recommended by the US FDA for devices that come into contact with the cardiovascular and lymphatic systems, whether directly or indirectly [18]. 

The original source material for the chitosan used in this research is shrimp shells, which contain calcium carbonate as the main mineral component of the exoskeleton matrix. Since ash content represents the amount of residual minerals in chitosan after processing from the original source material, the ash content is largely due to residual calcium carbonate minerals but may also contain other elements from the environment like lead and mercury that can have adverse effects on host cells and tissues [17]. The ash content of the starting chitosan material as well as for both the CM-films and TEA/tBOC-ESCMs was determined to be <0.5%. This low ash content is indicative of chitosan with a low amount of contaminating minerals. On the other hand, the Na_2_CO_3_-ESCM exhibited an ash content of 2.0 ± 0.6%, which was a significant four-fold increase over the other groups. This increase in ash content is attributed to residual Na_2_CO_3_ salts in or on the membrane after soaking in the highly concentrated Na_2_CO_3_ solution. Compared with other studies, the ash contents of the chitosan powders were generally less than 2% [19,20,21,22,23]. Hence, the ash contents of this study were within the range of other chitosan materials.

### 2.2. Fibroblast and Osteoblast Co-Culture 

The dual cell culture setup was an initial attempt to mimic the cell growth environment of periodontal treatment, aiming to investigate whether a chitosan membrane with a porous structure is superior to one without pores. The insert of the dual cell culture provided a relatively individual fibroblast growth environment, with the only communication pathway being the bottom membrane, facilitating the nutrient exchange between fibroblasts and osteoblasts. When the chitosan membrane fully covered the insert bottom, the membrane’s porosity determined the level of exchange. Despite the potential presence of chitosan extraction residues in all chitosan groups, the only variable across these groups was the presence of a porous structure in the membrane. A more porous membrane structure indicated a higher level of nutrient exchange between the two cell growth environments.

Focused on the aim of this study, which was to investigate whether a chitosan membrane with a porous structure was superior to one without pores, the selection of groups was aimed at minimizing variables. One control group consisted of samples without a chitosan membrane. This control was established to assess whether chitosan membranes effectively facilitated nutrient exchange, considering that the insert provides a clear pathway. The CM-film group was selected as another control to eliminate the potential influence of chitosan extraction residues and to focus on the variable of chitosan membrane porosity.

**Figure 2:**
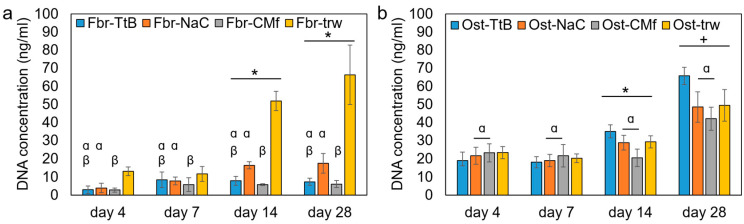
The dual cell culture results of (**a**) the top NIH 3T3 cell proliferation in the cell culture inserts and (**b**) the bottom MC3T3 cell proliferation on the well bottoms. * and + denote the significant difference in time points. α and β denote the significant differences among the cell culture groups. For abbreviations used in the graph, “Fbr-” indicates information regarding fibroblasts, while “Ost-” indicates information regarding osteoblasts. Regarding membrane types, “TtB” indicates the group with NIH 3T3 cells on the TEA/tBOC-ESCM, “NaC” indicates the group with NIH 3T3 cells on the Na_2_CO_3_-ESCM, “CMf” indicates the group with NIH 3T3 cells on the CM-Film, and “trw” indicates the group with NIH 3T3 cells on the insert with no membrane.

In the dual cell culture results, both the NIH 3T3 and MC3T3 E1 cells exhibited significant cell proliferation after 14 days, indicating that all the membranes were compatible with cell growth (Figure 2). In the previous study, Saos-2 osteosarcoma cells demonstrated significant proliferation after 5 days on both the TEA/tBOC-ESCM and the Na_2_CO_3_-ESCM [7]. Therefore, it can be inferred that the electrospun chitosan membranes and the cast film were compatible with both fibroblasts and osteoblasts.

The NIH 3T3 fibroblasts exhibited significantly more growth in the control inserts (without chitosan membrane/film) compared to the cells in inserts with chitosan membranes (Figure 2a). This difference is primarily attributed to the transwell polycarbonate membranes, which are specifically treated to promote cell attachment and growth. Significantly greater fibroblast growth was observed on the Na_2_CO_3_-ESCM membranes (Fbr-NaC) compared to the CM-film. However, no difference in growth was detected between the fibroblasts on the TEA/tBOC-ESCM (Fbr-TtB) and the other two membranes, suggesting that the difference was small. Regarding porosity, the TEA/tBOC-ESCM exhibited approximately 10 times greater porosity, while the CM-films had approximately 100 times less porosity than the Na_2_CO_3_-ESCM. This is consistent with previous research, indicating that higher porosity contributes to increased cell growth [24]. Moreover, the hydrophobic nature of chitosan membranes may have influenced fibroblast growth. The Na_2_CO_3_-ESCM group, being more hydrophilic, showed significantly higher growth compared to the CM-film group, which is more hydrophobic. This aligns with the theory that a more hydrophilic surface promotes increased cell growth [25]. 

The MC3T3 E1 cells in co-culture with the TEA/tBOC-ESCM exhibited significantly different cell growth as compared to other chitosan membrane groups (Figure 2b). It showed that the cell proliferation below the TEA/tBOC-ESCM was lower than the other membrane groups at day 4 and day 7. However, it showed higher cell proliferation than the other membrane groups after 14 and 28 days. At the same time, there was no significant difference between the Ost-TtB group and Ost-trw group, which were prior to the non-porous structure membrane groups. It suggested that the TEA/tBOC-ESCM did not have a negative effect on bone cell proliferation. Its porous structure, similar to the ECM structure, facilitated a nutrient exchange between the NIH 3T3 and MC3T3 E1 cell growth environments, potentially promoting long-term MC3T3 E1 cell proliferation.

**Figure 3:**
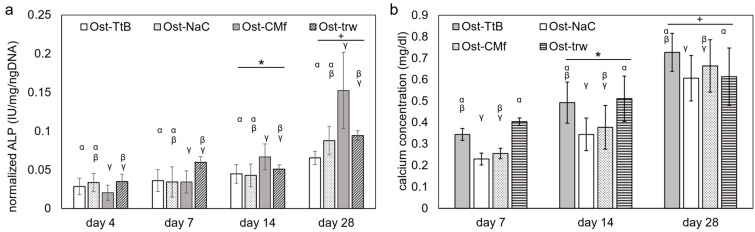
The dual cell culture results of (**a**) the ALP expression of the MC3T3 E1 cells and (**b**) the calcium deposit of the MC3T3 E1 cells at each time point. * and + denote the significant difference between day 4 and day 7. α, β and γ denote the significant difference among the cell culture groups. For abbreviations used in the graph, while “Ost-” indicates information regarding osteoblasts. Regarding membrane types, “TtB” indicates the group with NIH 3T3 cells on the TEA/tBOC-ESCM, “NaC” indicates the group with NIH 3T3 cells on the Na_2_CO_3_-ESCM, “CMf” indicates the group with NIH 3T3 cells on the CM-Film, and “trw” indicates the group with NIH 3T3 cells on the insert with no membrane.

The ALP concentration normalized to the DNA amount exhibited significant increases in all groups after 14 and 28 days (Figure 3a). Osteoblasts in co-culture with the fibroblasts on the CM-film showed the highest ALP activity of the test groups on days 14 and 28, while osteoblasts in co-culture with fibroblasts on the ESCMs tended to have ALP activities that were either comparable or lower than osteoblasts cultured alone or in control co-culture. However, the differences between these groups were not large since the osteoblasts cultured alone were not significantly different between all three membrane groups. It is further noted that ALP expression varies in a temporal fashion, and peaks in ALP expression may have been missed due to gaps between time points used. In a study by Ghuman et al., the ALP normalized to the control of the MC3T3 cells in the co-culture with gingival fibroblasts was significantly lower than the pure MC3T3 cell growth group [26], which implies that cross-talk between the fibroblasts and the MC3T3 cells might not stimulate ALP expression. In contrast, Zhu et al. showed that the relative ALP expression in MC3T3 E1 co-cultured with ephrinB2 transgenic periodontal ligament cells was significantly higher than in the pure MC3T3 E1 cell growth group [27]. Differences in these studies may be related to time points (day 3 vs. day 14) used to evaluate ALP in addition to differences in types of fibroblast cells [26,27]. Further research is still needed to explore the communication between fibroblasts and osteoblasts on osteoblastic differentiation and mineralization. Future studies may also be needed to consider the communication between gingival epithelial cells and osteoblasts. 

**Figure 4:**
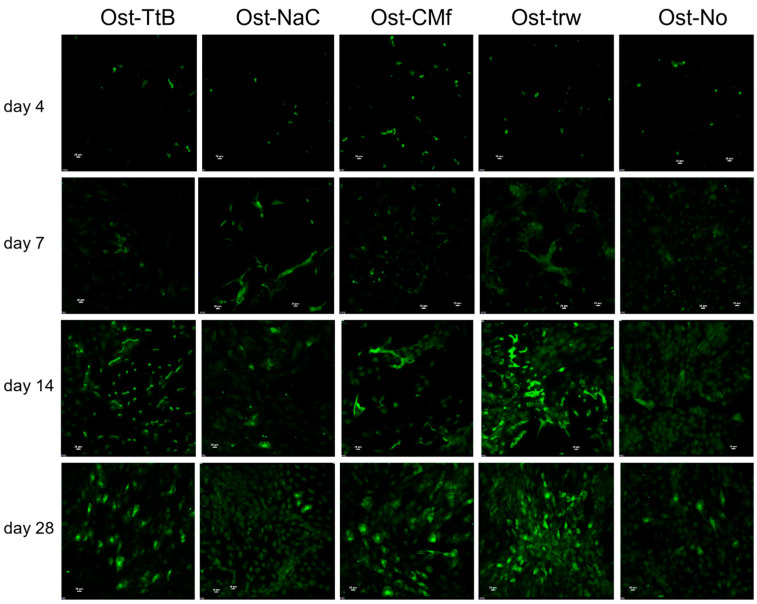
The fluorescent graphs of the anti-osteocalcin stained MC3T3 E1 cells at day 4, 7, 14 and 28. The osteocalcin increased from day 4 to day 28. For abbreviations used in the graph, while “Ost-” indicates information regarding osteoblasts. Regarding membrane types, “TtB” indicates the group with NIH 3T3 cells on the TEA/tBOC-ESCM, “NaC” indicates the group with NIH 3T3 cells on the Na_2_CO_3_-ESCM, “CMf” indicates the group with NIH 3T3 cells on the CM-Film, and “trw” indicates the group with NIH 3T3 cells on the insert with no membrane.

The amount of calcium, serving as an indicator of calcium-phosphate deposition, exhibited a significant increase in all groups after 14 and 28 days (Figure 3b), indicating cells were able to differentiate and elaborate a mineralized matrix. Among the groups, the Ost-TtB and the Ost-trw groups displayed a significantly higher calcium concentration than the Ost-NaCO_3_ group, while the Ost-CMf group showed a significantly lower calcium concentration than the Ost-trw group. The fluorescent stain graphs of osteocalcin (Figure 4) revealed increased production of osteocalcin after 14 days, serving as another indicator of heightened osteogenic activity similar to the calcium-phosphate deposition. The Ost-TtB group exhibited a significantly higher calcium concentration than the Ost-NaC group, suggesting that the TEA/tBOC-ESCM promotes cell mineralization better than the Na_2_CO_3_-ESCM. Although not statistically significant, the Ost-TtB group showed a trend of higher calcium concentration compared to the Ost-CMf group. Therefore, the TEA/tBOC-ESCM demonstrated similar or superior performance in cell mineralization compared to the CM-film. Since the TEA/tBOC-ESCM was the only type that preserved the fibrous structure, it could be inferred that the group with the nanostructure membrane showed improved support for osteogenic differentiation and elaboration of a mineralized matrix, which are important for regenerating bone. This supports the hypothesis that the porous electrospun membranes allow for better nutrient, small molecule and cell signal communication between the NIH 3T3 cells and the MC3T3 E1 cells. The higher calcium deposition observed in the Ost-trw group compared to the Ost-NaC and Ost-CMf groups further demonstrated the contribution of the mesh structure of the transwell, which provided optimal communication between the fibroblast and osteoblast growth environments. This suggests that the existing nutrient exchange between NIH 3T3 cells and MC3T3 E1 cells may have contributed to promoting calcium deposition. In Yang’s study, MC3T3 E1 cells showed more mineralization ability on the fibrous membrane with a higher chitosan content compared to the fibrous membrane with more PCL [28]. This suggested that chitosan itself may promote cell mineralization beyond the porous structure. In this study, the MC3T3 E1 cells were not directly in contact with the chitosan membranes, which could explain why chitosan did not significantly promote cell mineralization.

## 3. Materials and Methods

### 3.1. Chitosan Membrane and Film Preparation

Three types of chitosan-based membranes were prepared for this study: (a) TEA/tBOC-ESCM, (b) Na_2_CO_3_-ESCM and (c) a control CM-film treated with NaOH. The electrospun membranes were spun at 26 kV as described in previous studies using a 71% DDA chitosan (molecular weight = 311.5 kDa, Primex, Siglufjörður, Iceland) dissolved at 5.5 (*w*/*v*)% in 7:3 (*v*/*v*) trifluoroacetic acid (TFA, Thermo Fisher Scientific, Waltham, MA, USA) and dichloromethane (DCM, Sigma Aldrich, St. Louis, MI, USA). To make the TEA/tBOC-ESCM, membranes were first immersed in 10% (*v*/*v*) triethylamine/acetone solution for 24 h. After washing with acetone 3 times, membranes were immersed in the 0.1 g/mL tBOC solution for 48 h and then again washed with acetone 3 times [7]. Membranes were dried between two pieces of nylon mesh and under heavy weight to exclude the moisture in the air. To make the Na_2_CO_3_-ESCM, electrospun membranes were immersed in 5 M Na_2_CO_3_ solution for 3 h at room temperature and then dried between two pieces of nylon mesh [10].

To prepare CM-films, a 2 % (*w*/*v*) chitosan (71% DDA) in 2% acetic acid solution was prepared by stirring overnight. Twenty-eight millimeters of chitosan solution was pipetted into a petri dish (diameter = 8.5 cm) and allowed to dry for a few days at ambient conditions. For the neutralization, 2 M NaOH/DI H_2_O solution was pipetted into the petri dish to immerse films for 1 h. CM-film was rinsed with DI H_2_O for 30 min 3 to 5 times to remove excess NaOH and then dried in air at room temperature.

### 3.2. Membrane Characterizations

ESCM and solution-cast membranes were characterized for fiber structure and diameter, porosity, hydrophobicity, ash and endotoxin content. For fiber structure and diameter, specimens were examined using a scanning electron microscope (SEM, model EVO HD15, Carl Zeiss AG, Jena, Germany) from 2500× to 6000× after coating with 8 nm gold-palladium [7]. Fiber diameters were measured using image analysis software of the SEM. The porosity of test specimens was evaluated via porosimeter (Pascal 140 Mercury Porosimeter, Thermo Fisher Scientific, Waltham, MA, USA). Hydrophilic and hydrophobic character was measured by water contact angle using a VCA Optima measurement system (AST products, Inc, Billerica, MA, USA). Ash content of the test membranes was measured via combustion at 550 ± 20 °C to determine residual minerals from the original chitosan powder or as a result of the manufacture of ESCMs or the CM-films [29]. For endotoxin testing, gas-sterilized 1.5 cm diameter discs of test membrane samples were incubated in pyrogen-free water at a ratio of 1:100 (µg sample/µL water) for 24 h at 50 °C with constant shaking [30]. Endotoxin levels in pyrogen-free water extracts were determined using the Pierce LAL chromogenic endotoxin quantitation kit (Thermo Fisher Scientific, Waltham, MA, USA). For each material characterization method, three specimens of each test membrane type (*n* = 3/membrane) were evaluated. 

### 3.3. Fibroblast and Osteoblast Co-Culture 

Before the co-culture, the membranes underwent gas sterilization with ethylene oxide. The culture media was formulated by mixing α-MEM-media (Corning, Cellgro) with 5 mM β-glycerophosphate, 50 µg/mL ascorbic acid, 10 nM dexamethasone, 10% fetal bovine serum (FBS), 500 I.U./mL penicillin, 500 μg/mL streptomycin, and 25 μg/mL amphotericin-B. 

**Figure 5:**
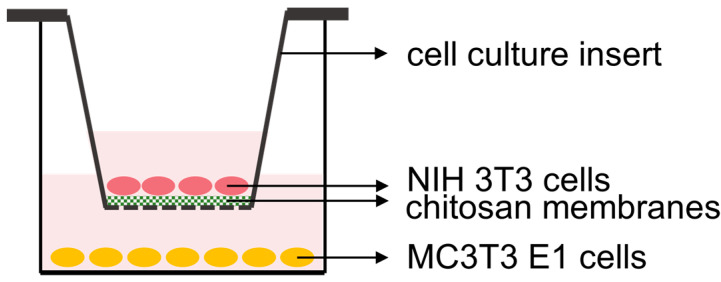
Diagram of co-culture arrangement of bone and fibroblast cells separated by a chitosan membrane. The MC3T3 E1 osteoblasts were seeded on the bottom of wells. After over-night incubation for osteoblast cell attachment, culture inserts containing a test chitosan membrane or no chitosan membrane (insert only) as a control were placed into the wells. Then, the NIH3T3 fibroblasts were seeded on the membrane/film samples.

Standard 24-well plates (Costar^TM^ flat bottom cell culture microplates, Corning, Glendale, AZ, USA) were used to seed osteoblasts and support inserts. First, MC3T3 E1 cells were seeded at 2 × 10^4^ cells/well of a 24-well plate in the medium and allowed to attach overnight. Medium was removed, and a Falcon^TM^ Transwell cell culture insert (pore size = 0.4 μm, Corning, Glendale, AZ, USA) was placed into each well. Next, a disc-shaped test or control chitosan membrane (diameter = 6 mm) was placed in the insert, and then fresh culture medium was added to the wells to cover the membranes. After about 24 h, the medium was removed, and 1 × 10^4^ NIH 3T3 cells/insert were seeded on either the test or control membranes and fresh medium was added to the wells to cover the cells in the wells and in the transwell inserts (Figure 5). 

The co-culture groups are described in Table 2. Cells were co-cultured for 28 days, with the medium being changed every 2–3 days. At days 4, 7, 14, and 28, osteoblast and fibroblast cell growth were evaluated using the Quant-iT^TM^ PicoGreen^TM^ dsDNA Assay kit (Thermo Fisher Scientific, Waltham, MA, USA) (*n* = 3/membrane or film/time point). The osteoblast cells were evaluated for alkaline phosphatase (ALP) enzyme activity as an early marker of bone cell differentiation using the QuantiChrome^TM^ Alkaline Phosphatase Assay Kit (BioAssay Systems) normalized to dsDNA, and mineral deposition on days 7, 14 and 28 via the Calcium Assay (Pointe Scientific, Inc., Canton, MI, USA) as a terminal indicator of differentiation. (*n* = 3/membrane or film/time point). For qualitative assessment of a late marker for bone cell differentiation (*n* = 1/membrane or film/time point), immunostaining of the bone cell matrix for osteocalcin was performed using a primary anti-osteocalcin antibody (BGLAP Picoband™, Boster Biological Technology, Pleasanton, CA, USA), followed by a Donkey anti-rabbit IgG ReadyProbes™ secondary antibody conjugated with Alexa Fluor 488 (Thermo Fisher Scientific, Waltham, MA, USA). Wells were then treated with ProLong™ Diamond Antifade Mountant with DAPI (Thermo Fisher Scientific, Waltham, MA, USA) prior to viewing on a Nikon Eclipse microscope (Nikon, Melville, NY, USA). Images were collected under the same brightness and contrast conditions to facilitate qualitative comparisons.

### 3.4. Statistical Analysis

The data for fiber diameter, water contact angle and ash content were analyzed by one-way analysis of variance (ANOVA) at the 0.05 level of significance. The cell culture data were analyzed by the two-way ANOVA (α = 0.05). As appropriate, Tukey’s post-hoc tests were used to distinguish significantly different groups at α = 0.05 level of significance.

## 4. Conclusions

In conclusion, the TEA/tBOC-ESCM preserved the nanofibrous and highly porous structure, whereas the Na_2_CO_3_-ESCM did not, and the CM-film did not have any fibrous structure and lacked porosity. Hence, the TEA/tBOC-ESCM had more pore volume than the other two. The CM-film was more hydrophobic than the other two electrospun membranes, and the TEA/tBOC-ESCM was more hydrophobic than the Na_2_CO_3_-ESCM. The ash contents of all the membranes were under 0.5% except for the Na_2_CO_3_-ESCM, which was under 2%. All the membranes/films had extremely low endotoxin concentrations, which were considered suitable for the FDA requirements. The in vitro evaluation showed that all the membranes were osteoblast- and fibroblast-compatible. The TEA/tBOC-ESCM showed more MC3T3 E1 cell proliferation and more or similar deposited calcium amount than the other two membranes/films. The higher amount of deposited calcium indicated faster mineralization with the TEA/tBOC-ESCM membrane. In clinical treatment, faster mineralization indicates faster bone formation. Hence, these results indicate that the highly porous nanofiber structure of the TEA/tBOC membranes may have an advantage in improving the bone healing/regeneration time in GBR clinical treatments.

## Figures and Tables

**Table 1:** Characteristics of TEA/tBOC-modified and Na_2_CO_3_-treated ESCM and solution-cast chitosan membrane films (CM-film). Data are expressed as mean (*n* = 3) ± standard deviation except for pore size, which is expressed as a median and percentiles due to a skewed pore size distribution.

	CM-Film	Na_2_CO_3_-ESCM	TEA/tBOC-ESCM
Median pore size (25th percentile, 75th percentile) (μm)	11.4 (4.7, 41.7)	12.2 (4.9, 42.5)	15.1 (6.6, 44.1)
Total pore volume (mm^3^/g)	0.1 ± 0.2 ^a^	32.4 ± 47.1 ^b^	461.0 ± 96.6 ^c^
Water contact angle (degree)	96.2 ± 2.4 ^a^	76.0 ± 10.8 ^b^	87.4 ± 9.0 ^b^
Endotoxin (EU/mL)	<0.1	<0.1	<0.1
Ash (wt%) ^&^	<0.5% ^a^	1.9 ± 0.5% ^b^	<0.5% ^a^

Letter superscripts indicate significant differences at α = 0.05 level of significance; ^&^ original chitosan powder ash content < 0.5%.

**Table 2:** Co-culture group descriptions.

Group	TEA/tBOC-ESCM	Na_2_CO_3_-ESCM	CM-Film	Control
Cell culture insert	NIH 3T3 cells on the TEA/ tBOC-ESCM (Fbr-TtB)	NIH 3T3 cells on the Na_2_CO_3_ -ESCM (Fbr-NaC)	NIH 3T3 cells on the CM-film (Fbr-CMf)	No membrane, NIH 3T3 cells only (Fbr-trw)
Well plate bottom	MC 3T3 (Ost-TtB)	MC 3T3 (Ost-NaC)	MC 3T3 (Ost-CMf)	MC 3T3 (Ost-trw)

## Data Availability

The data presented in this study are available on request from the corresponding author.
